# [^18^F]AV-1451 binding in vivo mirrors the expected distribution of TDP-43 pathology in the semantic variant of primary progressive aphasia

**DOI:** 10.1136/jnnp-2017-316402

**Published:** 2017-09-14

**Authors:** W R Bevan-Jones, Thomas E Cope, P Simon Jones, Luca Passamonti, Young T Hong, Tim D Fryer, Robert Arnold, Kieren S J Allinson, Jonathan P Coles, Franklin I Aigbirhio, Karalyn Patterson, John T O’Brien, James B Rowe

**Affiliations:** 1 Department of Psychiatry, University of Cambridge, Cambridge, UK; 2 Department of Clinical Neurosciences, University of Cambridge, Cambridge, UK; 3 Wolfson Brain Imaging Centre, University of Cambridge, Cambridge, UK; 4 Department of Pathology, Addenbrooke’s Hospital, Cambridge, UK; 5 Division of Anaesthesia, University of Cambridge, Cambridge, UK; 6 Cognition and Brain Sciences Unit, Medical Research Council Cognition and Brain Sciences Unit, Cambridge, UK

## Abstract

**Introduction:**

Semantic dementia, including the semantic variant of primary progressive aphasia (svPPA), is strongly associated with TAR-DNA binding protein 43 (TDP-43) type C pathology. It provides a useful model in which to test the specificity of in vivo binding of the putative tau ligand [^18^F]AV-1451, which is elevated in frontotemporal lobar degeneration tauopathies.

**Methods and results:**

Seven patients (five with svPPA and two with ‘right’ semantic dementia) and 12 healthy controls underwent positron emission tomography brain imaging with [^18^F]AV-1451. Two independent preprocessing methods were used. For both methods, all patients had clearly elevated binding potential (BP_ND_ (non-displaceable binding potential)) in temporal lobes, lateralising according to their clinical syndrome and evident in raw images. Region of interest analyses confirmed that BP_ND_ was significantly increased in temporal regions, insula and fusiform gyrus, consistent with those areas known to be most affected in semantic dementia. Hierarchical cluster analysis, based on the distribution of [^18^F]AV-1451 binding potential, separated semantic dementia from controls with 86% sensitivity and 100% specificity.

**Conclusions:**

[^18^F]AV-1451 binds in vivo regions that are likely to contain TDP-43 and not significant tau pathology. While this suggests a non-tau target for [^18^F]AV-1451, the pathological regions in semantic dementia do not normally contain significant levels of recently proposed ‘off target’ binding sites for [^18^F]AV-1451, such as neuronal monoamine oxidase or neuromelanin. Postmortem and longitudinal data will be useful to assess the utility of [^18^F]AV-1451 to differentiate and track different types of frontotemporal lobar degeneration.

## Introduction

The importance of biomarkers in neurodegenerative disorders is well established. Positron emission tomography (PET) has played an important role in biomarker development, illustrated by the impact of the Pittsburgh compound B on both research and clinical practice in Alzheimer’s disease.^1^ While beta-amyloid is central to the neuropathology of Alzheimer’s disease, in the vast majority of cases of frontotemporal lobar degeneration, the pathology is characterised by misfolding and aggregation of either tau (~40%) or TDP-43 (~50%), with fewer cases of fused in sarcoma pathology (<10%).^2–4^ A major aim for clinical research and drug development has been the development of biomarkers that enable pathological classification and longitudinal assessment in vivo, with quantitative and qualitative differentiation of neurodegenerative syndromes based on their underlying proteinopathy: tau versus TDP-43 versus beta-amyloid.

The radioligand [^18^F]AV-1451 was developed from compound screening in Alzheimer’s brains and is selective for tau versus beta-amyloid and versus alpha-synuclein.^5^ In vitro and in vivo studies have confirmed that [^18^F]AV-1451 binding in Alzheimer’s disease correlates strongly with phenotypical variation,^6^ clinical severity and Braak stage.^7–9^ There are fewer studies in frontotemporal lobar degeneration, but [^18^F]AV-1451 has also been shown to be sensitive in vivo to frontotemporal dementia associated with mutation of the microtubule-associated protein tau (MAPT) gene^10–12^ and in progressive supranuclear palsy.^13^ There is also evidence of modest binding to primary tauopathies in postmortem studies.^14^ However, the specificity of [^18^F]AV-1451 for tau pathology remains controversial.

Here, we test the properties of [^18^F]AV-1451 in semantic dementia, including the semantic variant of primary progressive aphasia (svPPA) and its non-dominant homologue, right semantic dementia (R-SD). Both syndromes display TDP-43 pathology in 75%–90% of cases,^15 16^ with tau pathology rarely present at postmortem. We test two complementary hypotheses:

1. In clinically diagnosed semantic dementia (likely TDP-43 pathology), the non-displaceable binding potential (BP_ND_) of [^18^F]AV-1451 is not increased compared with healthy older adults.
2. Independent of absolute levels of [^18^F]AV-1451 BP_ND_, the distribution of binding across brain regions is similar in patients and healthy controls.

## Methods

This study formed part of the Neuroimaging of Inflammation in Memory and Related Other Disorders study.^17^ The study protocol was approved by the UK National Research Ethics Service, East of England, Cambridge Central Research Ethics Committee (reference: 13/EE/0104) and the Administration of Radioactive Substances Advisory Committee. Five participants with svPPA and two with R-SD were recruited from the specialist clinic for frontotemporal dementia at the Cambridge University Hospitals National Health Service Foundation Trust. Participants were diagnosed according to published consensus criteria.^18^ Four of the seven participants had available biomarkers for Alzheimer’s pathology. Case L5 had a negative Pittsburgh compound B PET, whereas cases R3, L6 and L7 had cerebrospinal fluid beta-amyloid, total tau and ratio levels not supportive of Alzheimer’s disease. Clinical vignettes and neuropsychological test scores are summarised in table 1. Twelve similarly aged healthy participants acted as controls.

**Table 1:** Demographic, clinical, neuropsychological (ACE-R, FAB and PPT) and diagnosis for each participant with semantic dementia and for the group of 12 controls

**Case number**	**Demographics**	**Symptom duration**	**Presenting clinical features**	**ACE-R**	**FAB**	**PPT**	**Diagnosis**
L1	71, male, left-handed, 15 years of education	8 years	Anomia Impaired single-word comprehension Surface dyslexia Obsessional behaviour	43	11	25	svPPA
L2	69, male, right-handed, 9 years of education	7 years	Anomia Impaired single-word comprehension Surface dyslexia Inability to use previously familiar tools	9	3	0	svPPA
R3	59, male, right-handed, 14 years of education	6 years	Rigid obsessional behaviour Reduced empathy Prosopagnosia Anomia Impaired single-word comprehension Surface dyslexia	79	15	48	R-SD
R4	68, male, right-handed, 16 years of education	5 years	Rigid obsessional behaviour Reduced empathy Prosopagnosia Anomia Impaired single-word comprehension Surface dyslexia	77	11	28	R-SD
L5	64, female, right-handed, 14 years of education	4 years	Anomia Impaired single-word comprehension Surface dyslexia Prosopagnosia Concrete thinking	72	16	44	svPPA
L6	66, male, left-handed, 17 years of education	4 years	Anomia Impaired single-word comprehension Surface dyslexia Withdrawn behaviour Prosopagnosia	71	18	48	svPPA
L7	64, male, right-handed, 13 years of education	6 years	Anomia Impaired single-word comprehension Surface dyslexia Mild prosopagnosia	68	15	47	svPPA
Twelve controls	Age: 65.5 (range 55–74, SD 7.1) Sex: 6:6 Education years: 15.8 (range 11–19, SD 2.1)	NA	NA	95.3 (range 89–99, SD 3.2)	–	–	–

ACE-R, Addenbrooke’s Cognitive Examination—Revised; FAB, Frontal Assessment Battery; PPT, Pyramids and Palm Trees; R-SD, right semantic dementia; svPPA, semantic variant of primary progressive aphasia.

### MRI and PET imaging

Each participant underwent T1-weighted MRI (3T Siemens Trio or 3T Siemens Prisma, MPRAGE sequence, 1 mm isotropic voxels) before PET. Manufacture of [^18^F]AV-1451 used synthetic methods developed by Avid Radiopharmaceuticals, modified for GE TracerLab FX-FN synthesizer at the Wolfson Brain Imaging Centre, Cambridge. A GE Discovery 690 PET/CT scanner was used. A total of 370 MBq of [^18^F]AV-1451 was injected over 30 s at the onset of a 90 min scan. Emission data were reconstructed in 58 contiguous time frame images. Each emission frame was reconstructed using the PROMIS 3D filtered back projection algorithm into a 128×128 matrix 30 cm transaxial field of view, with a transaxial Hann filter cut-off at the Nyquist frequency.^19^ Corrections were applied for randoms, dead time, normalisation, scatter, attenuation and sensitivity. Each emission image series was aligned to correct for patient motion during data acquisition (www.fil.ion.ucl.ac.uk/spm/software/spm8). The BP_ND_ was determined by kinetic modelling with a simplified reference tissue model. The reference tissue was defined in the superior grey matter of the cerebellum, using a 90% grey matter threshold on the grey matter probability map produced by SPM8, smoothed to the PET resolution. The superior cerebellum was chosen as a reference region as it is unlikely to contain substantial pathology in semantic dementia (0 out of 15 cases of semantic dementia in the Cambridge Brain Bank had cerebellar pathology).

Two independent preprocessing methods were evaluated. (1) The data were coregistered with T1-weighted images. Regions of interest were defined by cortical parcellation and modified subcortical segmentation using the Desikan-Killiany atlas in the PetSurfer toolbox within Freesurfer.^20 21^ The BP_ND_ values in each region were partial volume corrected using the symmetric geometric transfer matrix method.^22^ (2) The mean aligned PET image, and hence the corresponding aligned dynamic PET image series, was rigidly registered to the T1-weighted image using SPM8, so as to extract values from the Hammersmith atlas n30r83 (http://brain-development.org/brain-atlases) modified with brainstem and cerebellar parcellation. All region of interest data, including the reference tissue values, were corrected for the cerebrospinal fluid fraction through division with the mean region of interest probability (normalised to 1) of grey plus white matter segments, each smoothed to PET resolution.

### Analysis

To test hypothesis 1, we used general linear models with t-tests for each region of interest, excluding extraparenchymal regions, first comparing each patient to the control group, as a case series (cf Bevan Jones *et al* 10). We then compared the patients groupwise to the controls. Data were corrected for multiple comparisons to control the false discovery rate (FDR) at q<0.05.^23 24^

To test hypothesis 2, regional binding potentials for each subject were converted to a linear vector. Spearman’s rank order method was used to perform non-parametric correlation with all other subjects, creating a correlation ‘similarity’ matrix of the distribution of BP_ND_, disregarding its absolute intensity. The inverse of this matrix (the ‘dissimilarity’ matrix) formed the input to multidimensional scaling and hierarchical cluster analysis. The performance of both farthest neighbour and average linkage methods was assessed (cf Passamonti *et al* 13).

## Results

**Figure 1:**
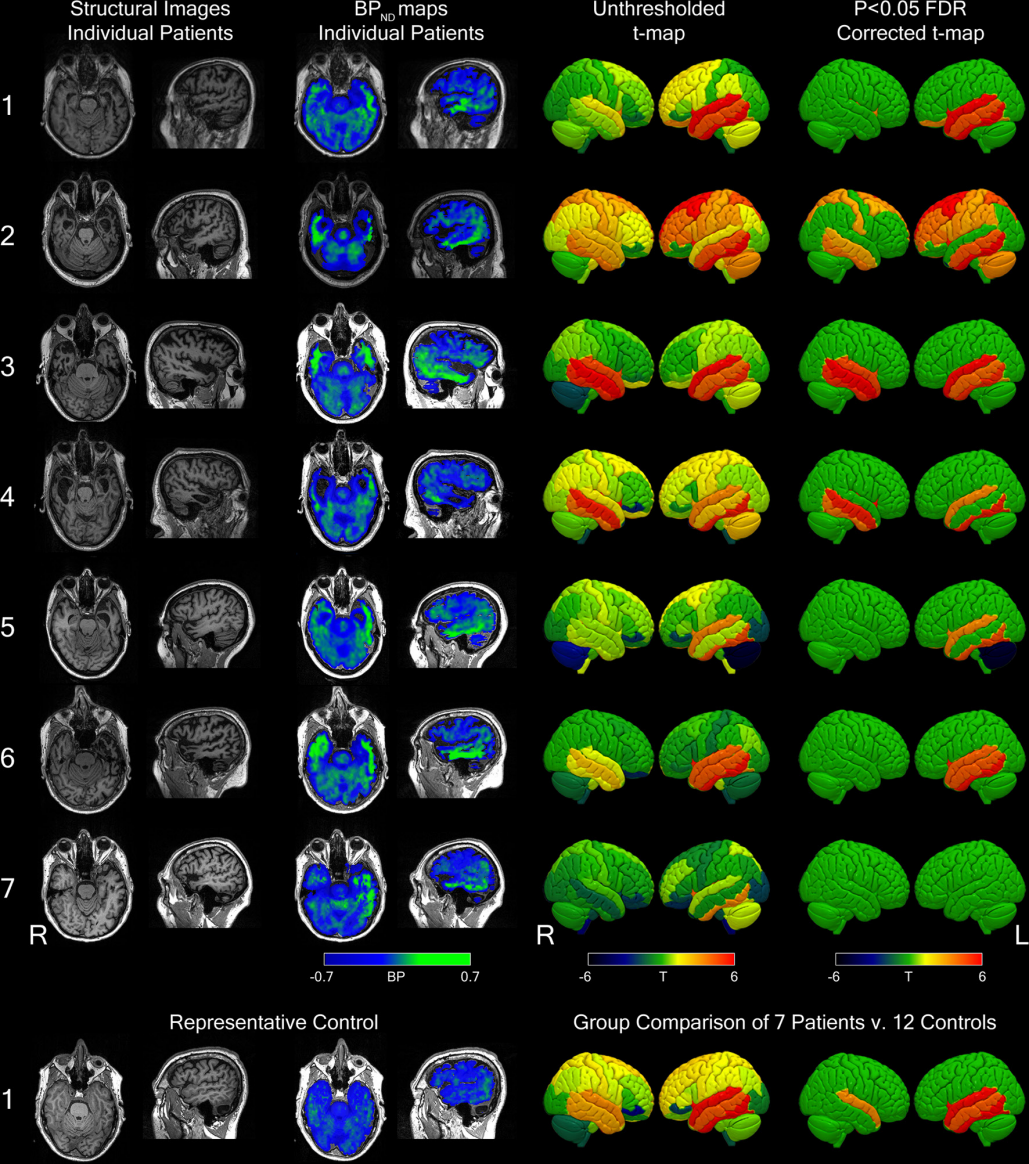
Using data preprocessed by method 1. (Upper panel) Column 1: axial and sagittal views of T1-weighted images for each semantic dementia participant, column 2: raw axial and sagittal BP_ND_ maps for each patient, column 3: unthresholded surface-rendered regional T-maps for each subject against all controls and column 4: equivalent T-maps thresholded at q<0.05, corrected for false discovery rate. (Lower panel) T1-weighted images and BP_ND_ maps for a representative control and the group comparisons of all 7 patients versus 12 controls both uncorrected (column 3) and corrected for false discovery rate q<0.05 (column 4). The numbering of individual patients is consistent with figure 2 and table 1. BP_ND_, non-displaceable binding potential. FDR, false discovery rate.

Figure 1 shows the axial and sagittal views of the T1-weighted images for each participant with semantic dementia, confirming the severe asymmetric temporal polar atrophy in svPPA and R-SD, respectively. Also shown are the uncorrected raw [^18^F]AV-1451 BP_ND_ maps for each individual patient and a representative control. Statistical comparisons against controls using method 1 are shown for each patient individually and for the group. All patients had significant elevation of BP_ND_ in temporal lobes compared with controls, and in all but one individual, this survived correction for multiple comparisons across the whole brain. As a group, for method 1, significant elevation of BP_ND_ at false discovery rate (FDR q<0.05 was observed in the following regions of the left hemisphere: superior, middle and inferior temporal lobes; insula cortex; fusiform gyrus; temporal banks and accumbens. In the right hemisphere: amygdala, caudate and superior temporal cortex. For method 2, the following regions showed significant elevation of BP_ND_ at FDR q<0.05. In the left hemisphere: medial anterior and lateral anterior temporal lobes; superior, middle and inferior temporal gyri; fusiform gyrus; insula; thalamus and nucleus accumbens. In the right hemisphere: lateral anterior temporal lobe; middle and inferior temporal gyri and medial anterior temporal lobe.

**Figure 2:**
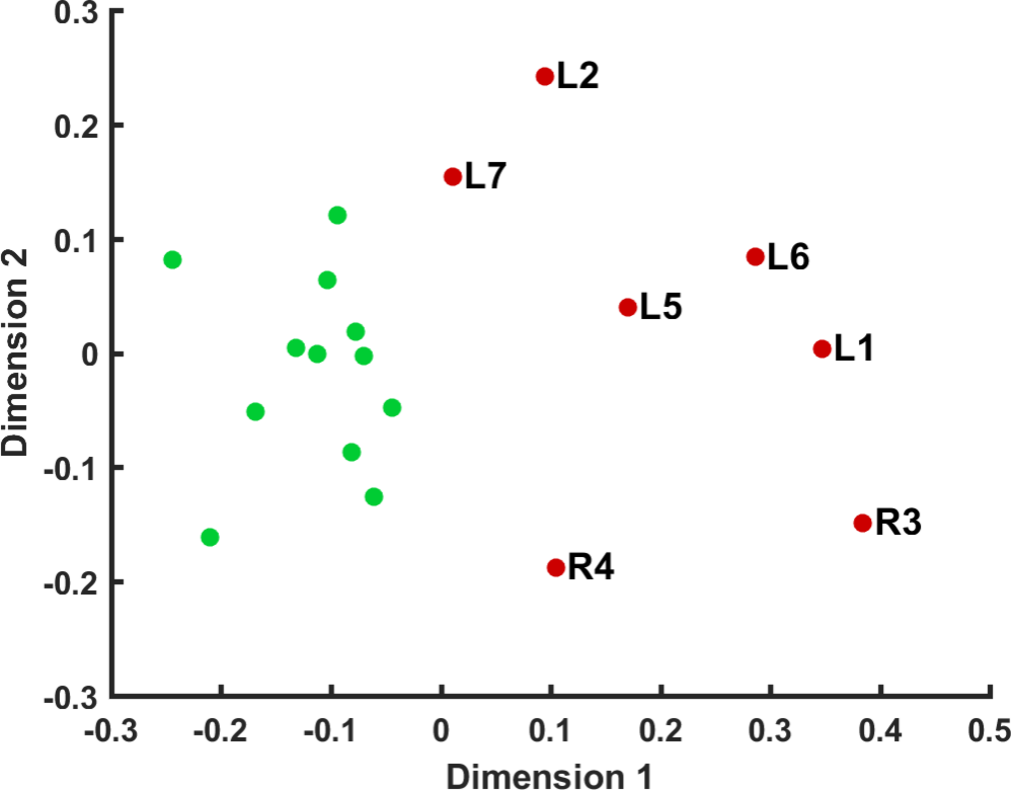
Two-dimensional scaling of non-parametric whole brain regional correlations using data preprocessed by method 1. Red dots represent patients, and green dots controls. Patient labels correspond to individuals in table 1.

Non-parametric multidimensional scaling of BP_ND_ distribution clearly separated patients from controls (figure 2). With either preprocessing method, hierarchical cluster analysis of these data detected SD with 86% sensitivity and 100% specificity. Cluster analysis, blinded by non-parametric methods to the degree of ligand binding, therefore provided statistically significant unsupervised classification (Yates’ corrected χ^2^(1, n=19)=11.3, p=0.0008). Identical results were obtained with both average and farthest neighbour linkage methods. The patient misclassified as a control had mild and relatively early disease and lay between the control and patient distributions on multidimensional scaling (figure 2, case L7).

## Discussion

We present evidence for consistently elevated [^18^F]AV-1451 BP_ND_ in seven cases with either svPPA or its non-dominant right hemisphere homologue, R-SD. Contrary to the null hypotheses, in all seven cases individually and at the group level, the regions known to be most affected by TDP-43 pathology^25 26^ demonstrated increased [^18^F]AV-1451 binding compared with controls. This did not merely reflect a global increase in BP_ND_, as six of the seven cases were correctly classified by the relative *distribution* of BP_ND_ across the brain. The significant regional binding to likely TDP-43 pathology in svPPA and R-SD indicates that this ligand is not selective for tau and casts doubt on the utility of [^18^F]AV-1451 to subtype frontotemporal dementias according to tau versus TDP-43 pathology. While this suggests that the use of [^18^F]AV-1451 to select tau-mediated frontotemporal dementia populations for clinical trials is unlikely to be effective, the ligand may still retain a potential role in the longitudinal assessment of the degree and distribution of pathological burden across the frontotemporal dementia spectrum.

Semantic dementia in the form of svPPA represents a highly stereotyped and well-defined clinical syndrome with characteristic structural neuroimaging.^27–29^ Its non-dominant homologue displays similar features but typically with a later presentation, more behavioural disturbance and prosopagnosia.^30^ Within the spectrum of frontotemporal dementias, both svPPA and R-SD show very strong clinicopathological correlations with TDP-43 pathology, especially type C.^4 15 25 26 31^ This contrasts with the behavioural variant of frontotemporal dementia which can result from either TDP-43 or tau pathology with approximately equal likelihood.

Although the majority of semantic dementia cases have TDP-43 pathology, a small minority of cases may arise from tauopathies, most often Alzheimer’s disease pathology or Pick’s disease pathology.^15 16 31^ In keeping with this, 25 of 34 patients clinically diagnosed with semantic dementia (or svPPA) and in the Cambridge Brain Bank are positive for ubiquitin or TDP-43 and negative for both tau and beta-amyloid. Similarly, the recent report of postmortem examination in clinical cases of svPPA at four centres in North America^16^ confirmed that the majority (24/29) had TDP-43 pathology (mainly type C). The five who did not have TDP-43 pathology exhibited clinical features out of keeping with the classical syndrome of svPPA, such as short disease duration and extrapyramidal features. These features are not present in any of the individuals we report. Although we do not have autopsy confirmation of the cases reported here, four out of seven participants have negative biomarkers for Alzheimer’s pathology and, overall, it is highly unlikely that the explanation for our findings is that all individuals in our study had a primary or secondary tauopathy.

While the in vivo and postmortem studies of [^18^F]AV-1451 in Alzheimer’s disease are compelling, the binding characteristics of [^18^F]AV-1451 in non-AD tauopathies remain controversial. In vivo studies have been encouraging, with significant binding demonstrated in frontotemporal dementia due to mutations in the MAPT) gene^10 12 32^ and in progressive supranuclear palsy.^13^ This contrasts with reports from postmortem studies, which predominantly describe low level binding to the tau aggregates of frontotemporal lobar degeneration.^14 33 34^ These postmortem studies make it increasingly clear that the primary, tertiary and quaternary structures of tau, as well as the type and maturity of tau pathology,^34^ are important determinants of [^18^F]AV-1451 binding. This implies that the predominantly straight filaments of 4-repeat tau that constitute the pathology of progressive supranuclear palsy and corticobasal degeneration, and the 3-repeat tau of intraneuronal Pick bodies, lead to less intense binding than that seen in Alzheimer’s pathology, with its balanced 3-/4-repeat tauopathy in the form of paired helical filaments.

The discordance between in vivo and postmortem findings led to the proposal of ‘off target’ binding sites. In the last 2 years, several possibilities have been hypothesised, particularly to explain the characteristic pattern of basal ganglia binding seen in almost all participants. These include neuromelanin,^33 35^ iron, calcium and Biondi ring tangles.^34^ None of these potential targets is anatomically compatible with the pattern of cortical binding seen here, which is in a distribution expected for pathology in semantic dementia.^25 31^ One plausible explanation for the elevated signal observed here could be spill out from increased binding in white matter, for example, to the expression of monoamine oxidase B by reactive astrocytes. The intrinsic resolution of PET, combined with the degree of atrophy in the semantic dementia cohort, makes it very difficult to distinguish binding in white or grey matter. However, there is a paucity of evidence for [^18^F]AV-1451 binding to monoamine oxidase B, unlike other tau radioligands such as the THK compounds. The possibility of binding to monoamine oxidase B was explored early in the development of [^18^F]AV-1451,^5 36^ and its favourable profile in this regard was important in its progress to clinical studies.

Another possibility is that [^18^F]AV-1451 binds to very low levels of abnormal tau that have been reported to coexist with TDP-43 in some cases,^25^ to proteins associated with cellular stress in TDP-43 pathologies or to some other cellular marker of neurodegeneration. Alternatively, it could be that the in vivo binding demonstrated here mirrors the postmortem binding of [^18^F]AV-1451 to TDP-43 type C,^14 34^ despite the low level or absent binding to most TDP-43 pathology.^14 34^ Overall, we retain an open mind as to the identity of the non-Tau proteins and cell types to which [^18^F]AV-1451 is binding in semantic dementia.

One must also consider some caveats in the analytical methods of the imaging pipelines. In particular, the extreme regional atrophy of semantic dementia complicates normalisation and PET analysis, including modelling decisions such as partial volume correction, which is necessary in order to prevent significant binding being obscured by the degree of atrophy. Misregistration errors arising from extreme atrophy may also influence PET estimates. However, these considerations are unlikely to account for our findings for two main reasons. First, the bright signal of elevated [^18^F]AV-1451 binding is visible in the temporal lobes of uncorrected BP_ND_ maps in all single subjects in native space (figure 1). Second, highly similar patterns of significant binding are seen with two independent methods of data preprocessing, using different tissue segmentation and correction methods and parcellation with different brain atlases. The use of an appropriate reference tissue region may also be complicated in neurodegenerative diseases. In particular, there is emerging evidence that specific patterns of atrophy occur in the cerebellum across a range of disorders.^37^ In frontotemporal dementia, cerebellar atrophy and pathology are well described in cases of the behavioural variant and particularly in cases resulting from expansions in C9orf72.^38^ However, in semantic dementia, cerebellar atrophy has not been described, and the typical distribution of TDP-43 type C pathology does not involve the cerebellum.^39^ We have examined 15 cases of semantic dementia in the Cambridge Brain Bank, and in no case was cerebellar pathology found. Additional reassurance that our results are not driven by a possible group difference in cerebellar pathology comes from our hierarchical cluster analysis. This non-parametric analysis of the distribution of pathology across the whole brain is blind to absolute BP_ND_ values; the effect of a group difference in a reference region would be to change the overall level of binding across the brain, without modifying the relative distribution of pathology. The fact that we were able to recover the group structure with 86% sensitivity and 100% specificity argues against cerebellar pathology being a significant driver of our findings.

Validation of the specificity of [^18^F]AV-1451 binding in both Alzheimer’s disease and frontotemporal lobar degeneration is highly important. Binding in the presence of neurodegeneration without tauopathy poses serious questions for both clinical and research applications of this ligand, although it may nonetheless be useful to evaluate the progression of neurodegenerative diseases and normal ageing. The magnitude of elevations in binding potential was similar to those previously observed in MAPT mutation^10^ and progressive supranuclear palsy, but lower than those observed in Alzheimer’s disease.^13^ While this study did not directly compare FTD-TDP (in semantic dementia) to FTD-tau cases, the lack of selectivity of [^18^F]AV-1451 for tauopathies challenges the utility of this ligand for pathological differentiation in vivo. Determining the binding site or sites will be important as, even if this is not specific to tau aggregation, it may provide valuable insights into the cellular mechanisms of neurodegeneration, perhaps in regions that are yet to display volume loss or hypometabolism. In order to determine the best use of this ligand, full characterisation of the behaviour of [^18^F]AV-1451 in frontotemporal lobar degeneration with longitudinal imaging and postmortem validation is essential.
